# Performance Analyses of Energy Detection Based on Square-Law Combining in MIMO-OFDM Cognitive Radio Networks

**DOI:** 10.3390/s21227678

**Published:** 2021-11-18

**Authors:** Josip Lorincz, Ivana Ramljak, Dinko Begušić

**Affiliations:** 1 Faculty of Electrical Engineering, Mechanical Engineering and Naval Architecture (FESB), University of Split, 21000 Split, Croatia; dinko.begusic@fesb.hr; 2 Elektroprenos–Elektroprijenos BiH” a.d. Banja Luka, 88000 Mostar, Bosnia and Herzegovina; ivana.ramljak@elprenos.ba

**Keywords:** energy detection, spectrum sensing, cognitive radio networks, OFDM, MIMO, SLC, detection, false alarm, probability, SNR, transmit, receive, wireless, antennas, modulation, power

## Abstract

Cognitive radio (CR) technology has the potential to detect and share the unutilized spectrum by enabling dynamic spectrum access. To detect the primary users’ (PUs) activity, energy detection (ED) is widely exploited due to its applicability when it comes to sensing a large range of PU signals, low computation complexity, and implementation costs. As orthogonal frequency-division multiplexing (OFDM) transmission has been proven to have a high resistance to interference, the ED of OFDM signals has become an important local spectrum-sensing (SS) concept in cognitive radio networks (CRNs). In combination with multiple-input multiple-output (MIMO) transmissions, MIMO-OFDM-based transmissions have started to become a widely accepted air interface, which ensures a significant improvement in spectral efficiency. Taking into account the future massive implementation of MIMO-OFDM systems in the fifth and sixth generation of mobile networks, this work introduces a mathematical formulation of expressions that enable the analysis of ED performance based on the square-law combining (SLC) method in MIMO-OFDM systems. The analysis of the ED performance was done through simulations performed using the developed algorithms that enable the performance analysis of the ED process based on the SLC in the MIMO-OFDM systems having a different number of transmit (Tx) and receive (Rx) communication branches. The impact of the distinct factors including the PU Tx power, the false alarm probability, the number of Tx and Rx MIMO branches, the number of samples in the ED process, and the different modulation techniques on the ED performance in environments with different levels of signal-to-noise ratios are presented. A comprehensive analysis of the obtained results indicated how the appropriate selection of the analyzed factors can be used to enhance the ED performance of MIMO-OFDM-based CRNs.

## 1. Introduction

Due to the huge enlargement in the number of new applications and the constant demand for faster data rates in modern wireless communication systems, the radio frequency (RF) spectrum has become crowded. For a large part of the available spectrum, regulatory bodies have already granted licenses for exclusive spectral use. This makes the development and expansion of new services in wireless networks challenging. Previous analyses indicated that the lack of flexibility in the spectrum access represents a significant problem since many licensed spectrum portions are not effectively used. This lack of RF spectrum in wireless communication has prompted the development of a new generation of devices that will be aware of their RF environment and enable the flexible and effective usage of the available spectral resources [1,2,3].

Cognitive radio (CR) technology as a candidate for upcoming communication systems represents a potential solution that leads to the more efficient usage of the spectrum. CR enables unlicensed/secondary users (SUs) to have dynamic spectrum access (DSA) to licensed frequency bands, when these bands are underutilized/unutilized by their licensed/primary users (PUs). CR, therefore, enables the exploitation of licensed bands in periods when there is no PU activity, meaning that SU should have no influence on the transmission efficiency of the PU [4,5,6,7].

**Figure 1:**
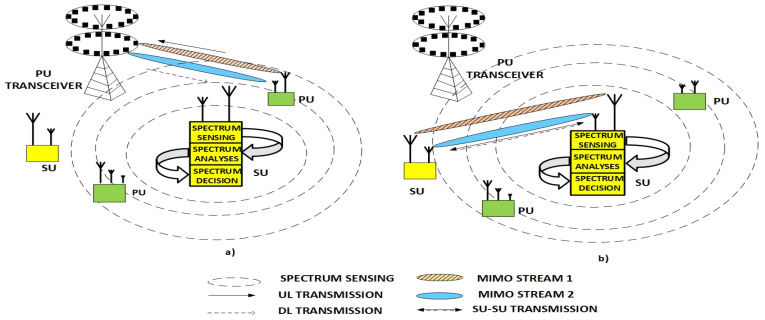
Operation of cognitive radio in MIMO communication systems for the case when (**a**) SU detects spectrum occupied by PU and (**b**) SU performs transmission due to detection of spectrum unoccupied by PU.

Figure 1a,b illustrates the concept of CR for the case when the frequency spectrum is occupied and unoccupied by the PU transmission, respectively. The main process in CR is related to spectrum sensing, followed by spectrum analyses and deciding about spectrum occupancy. Although different local spectrum-sensing (SS) methods have been proposed in the existing literature, energy detection (ED) as one of the broadly accepted local spectrum-sensing method is analysed in this work due to the many advantages that such a method involves. More specifically, the ED method is a basic non-coherent and non-cooperative method and one of the most used local SS techniques in practice. The main advantages of the ED method compared to other local SS methods lie in its low computational complexity, low application cost, simple implementation, and semi-blind process, which eliminates the need for prior information about the PU signal. Such advantages make the ED method a promising candidate for implementation in different applications and devices, especially those in which operation is based on limited battery power supply such as sensors and mobile devices. Due to the proliferation of multi-antenna sensors and mobile devices, the examination of the performance of the ED method can give important cognitions in terms of the practical implementation of the ED method in such communication environments. This motivates a deeper investigation of ED performance in multi-antenna communication systems.

Besides the many advantages of the ED method, the main challenge for the accurate detection of the PU using the ED method is the necessity of having exact information about the noise power estimation at the precise position of the SU. Obtaining the exact noise power estimation is not always possible due to challenges such as filtering effects, the interference caused by other signals, and thermal noise. This lack of exact knowledge about the noise power can cause a problem known as noise uncertainty (NU), which consequently results in PU signal detection errors. It is also known that ED has a poor level of performance when the PU signal detection is performed for signals below a certain value of signal-to-noise ratio (SNR) level known as the SNR wall. In addition, the inability to distinguish among a PU signal, SU signal, and interference is also one of the disadvantages of ED methods [8,9,10].

Despite the presented drawbacks of the ED method, due to its simple implementation, ED as an SS method is one of the most frequently used methods in practice. When ED is used for SS, the received signal is detected as a measure of energy in a particular part of the spectrum. The energy detector of SU compares the estimated energy with a threshold value to decide on the presence or absence of the PU signal. Dynamic threshold (DT) adaptation and traditional fixed threshold approaches are two main selection criteria for setting the detection threshold of the PU signal in the ED process [11,12,13,14]. Due to the NU, the detection process with a fixed threshold becomes unreliable. The techniques based on DT adaptation can handle problems with signal fluctuations in situations where the noise power impacts on the SS. Consequently, such an approach can gain better results in terms of the PU detection efficiency [15,16].

One of the main characteristics of cognitive radio is the possibility of a dynamic adaptation to the propagation environment. Orthogonal Frequency-Division Multiplexing (OFDM) is a multicarrier modulation technique that can prevent many of the propagation drawbacks that occur in high-bit-rate communications. The main advantage of OFDM is its capability for adaptation to wireless channel variations. OFDM also implements a cyclic prefix (CP) to reduce the inter-symbol interference (ISI) and mitigate multipath fading. Due to these capabilities, OFDM is considered to be a transmission technique that can fulfill the requirements of cognitive radio networks (CRNs) [17,18,19].

Besides the usage of the OFDM technique, the implementation of systems based on a combination of OFDM and Multiple-Input Multiple-Output (MIMO) transmissions have gained popularity in the last few years. This is because MIMO improves the spectral efficiency, reliability, quality of service (QoS), capacity, and data throughput of wireless communication networks. Before the introduction of MIMO, single-input-single-output (SISO) systems were predominantly used. SISO systems have a very low throughput and cannot support a large number of users with high reliability [20]. MIMO addresses these issues by “combining” signals on the branches (antennas) of the transmitting and the receiving devices. This improves the quality of the transmission and the users’ data rate [21].

In a MIMO system, each antenna element operates on the same frequency and does not require additional bandwidth for enlarging the transmission capacity. MIMO systems also require less transmit (Tx) power than SISO systems for transmission in the same radio environment. The main advantages of MIMO systems include the possibility of implementing beamforming and obtaining transmission gains through spatial diversity and multiplexing [21,22].

Since spectrum congestion has become a significant problem, the MIMO concept has been put forward as a technique that can contribute to the improvement of spectral efficiency in communication systems [23,24,25,26,27,28]. By combining MIMO transmissions and OFDM, high spectral efficiency and strong interference resistance can be achieved [29]. If combined with beamforming in CRNs, the MIMO technique can offer a potential solution to the practical realization of dynamic spectrum access [23,24]. In [30,31], it was shown that MIMO systems can increase the probability of PU detection and decrease both the probability of PU misdetection and false alarms in the SS process.

The SS of MIMO-OFDM signals can be achieved through the exploitation of diversity combining techniques. The most prominent diversity combining techniques are Maximal Ratio Combining (MRC), Equal Gain Combining (EGC), and Square-Law (SL) techniques [32]. To perform non-coherent energy detection, the MRC and EGC techniques request full or partial channel state information (CSI), respectively. Compared with non-coherent ED based on SL techniques such as Square-Law Combining (SLC) and Square-Law Selection (SLS), the MRC and EGC techniques have significantly more complexity when implementing SS. Since non-coherent combining schemes can exploit the diversity gain in the absence of CSI, they represent a simpler solution for practical implementation of the ED method. Such simplicity in the practical implementation of the SLC technique was additional motivation to develop and present in this paper a simulation study that enables the evaluation of SS performance based on the ED of signals in MIMO-OFDM systems.

Hence, the implementation of the MIMO-OFDM transmission can increase the robustness to the NU of the energy detection process. Due to these performance characteristics, for this paper, the ED in the systems combining MIMO-OFDM transmissions was analysed and the exploration of the SLC ED performance in the appropriate CRNs was performed. The main contributions of this paper include:

- The development of a new mathematical formulation for the ED of MIMO-OFDM signals based on the SLC method;
- The introduction of developed algorithms for simulating ED performance using SLC for the signals transmitted in MIMO-OFDM systems with different combinations of PU (Tx) and SU (Rx) branches (antennas); and
- The investigation of the impact of different parameters such as Tx powers, modulation types, the number of samples used for ED, the level of false alarm probability, and the number of MIMO Tx-Rx branches (antennas) on the detection probability of the signals detected using the SLC ED method in MIMO-OFDM systems.

The rest of the paper is organized as follows. Section 2 gives an overview of the topic related to the ED of the signals transmitted in MIMO-OFDM systems. The mathematical model expressing the relationship among parameters used in the analysis of the ED performance is presented in Section 3. The algorithms developed for simulating the signal transmission and the ED process in the case of symmetric and asymmetric MIMO-OFDM systems impacted by NU variations and DT adaptations are presented in Section 4. In Section 5, the evaluation and extensive explanation of the results obtained through the simulations is presented. Finally, the concluding remarks are given in Section 6.

## 2. Related Works on the Spectrum Sensing of OFDM Signals Based on the ED Method

The previous studies demonstrated that MIMO and OFDM are widely accepted as key enabling technologies in CR systems [33,34,35,36,37]. It was shown in [36,37] that CR in MIMO-OFDM systems using ED schema for SS offers a potential improvement in detection performance.

In [36], an analysis was performed with a focus on detecting PU signals in MIMO systems by utilizing the SLC ED method. In comparison with other ED methods, ED based on SLC has a better detection performance in low and moderate SNRs and for a higher number of OFDM symbols. In [37], a detailed analysis of SS for multiple antenna transmission techniques is given. The impact of the different modulation schemas, symbol lengths, false alarm probabilities, and SNRs on the detection probability in OFDM ED CRNs was analyzed. A simulation was performed for single-input multiple-output (SIMO) and single-input single-output (SISO) systems. The results indicated that the EGC method offers the highest gain in terms of ED precision, but, on the other hand, it requires perfect CSI. The SLC method provides a lower gain in terms of ED precision, but it does not require any prior CSI. However, the SLC method requires several detectors and combiners, which increase the implementation cost. The overall results indicated that a longer OFDM symbol length refers to a better detection probability.

Considering the low complexity and versatility of the ED techniques, besides the SLC technique, the performance of SLS antenna diversity techniques at the SU was analyzed in [38]. That paper addressed the challenges involved in the hardware deployment of ED systems exploiting SL diversity techniques. ED in terms of hardware deployment was analyzed in [38] with a focus on challenges such as modeling the SNR estimation, radio-frequency imperfections, PU signal modeling, and realizing the effects of fading channels. Solutions based on simplifications have been proposed to ensure the hardware deployability of SL diversity techniques in realistic scenarios.

The performance analysis of the ED method using receiver operating characteristic (ROC) curves for various fading channels is detailed in [39,40,41,42]. In [39], the SLC diversity reception, also known as the soft decision fusion scheme, was evaluated with a focus on the Additive White Gaussian Noise (AWGN), Rayleigh, Rician, Nakagami, Weibull, and Hoyt fading channels. The performance comparison of the soft decision fusion scheme was analyzed with a focus on network parameters such as the number of CRs in the network, the time–bandwidth product, and the average SNR. The results indicated that the performance of ED is not degraded significantly in low and moderate shadowing conditions. Implementing diversity in the detection of PU signals mitigates the effect of fading in the detection performance. In [40], the problem of ED of an unknown signal over a multipath channel is demonstrated. The authors presented alternative closed-form expressions related to the probability of detection in the Rayleigh and Nakagami fading channels. The results of the simulations indicated improvements in the detection capability for relatively low-power applications, when multiple antenna transmission techniques are used.

A tutorial presenting different ED methods and the basic parameters of the classical energy detector in SISO transmission systems is given in [41]. For ED based on two SL diversity schemes (SLS and SLC), the mathematical formulation of false alarm and detection probability in MISO transmission systems is given. Alternative ED methods such as a double-threshold energy detector, P-norm detector, and energy detection for full-duplex nodes are also described.

In [42], the concept of continuous SS based on ED at the base station (BS) in the environment with an arbitrary number of PUs, SUs, and sensors at the BS was analyzed. Based on the proposed theoretical approximations, mathematical expressions for the detection and false alarm probabilities were developed and the analysis of the ED performance for a large number of samples used for detecting Gaussian signals was performed. The results show how an increase in the number of sensors on the receiver side (BS) contributes to the improvement of the detection efficiency.

Furthermore, higher detection probabilities of a PU signal can be accomplished by cooperative spectrum sensing (CSS) and SLC, as shown in [9,39,43]. In CSS, the information from different CR users is combined to make a decision. CSS together with multiple antenna techniques (such as SLC) results in the better detection performance of OFDM signals at low values of SNR [43]. The author in [9] showed that diversity combining schemes can help to reduce the impact of multipath fading. The obtained results showed that an increase in the multipath and/or shadowing parameters led to improved detection performance.

Since the performance of ED is limited by the NU, the implementation of a kernelized ED approach with a detection threshold was presented in [27]. ED was performed for PU signals distorted by Gaussian mixture noise in Internet of Things’ (IoT) networks. The results indicated that the detection performance of kernelized ED can be enhanced if the SNR, the number of samples, and the number of Rx antennas are increased.

Authors in [44] proposed an algorithm for signal identification in Space-Time Block Code (STBC)—OFDM communication systems based on exploiting the cross-correlation as a discriminating feature of the received signals from two antennas. In [45], the authors extended the possibility of implementing the proposed algorithm on multi-antenna systems at the Rx side. Compared to the ED method, the main advantage of the proposed signal identification is that it does not require exact knowledge about noise power for performing signal identification. However, computation complexity, identification time in case of STBC-OFDM systems with a larger number of Rx antennas, and limitation related to the inability of identifying signals transmitted using other coding schemes besides STBC significantly limit the practical implementation of this method in comparison with the ED method.

In [46,47], we analysed the impact of NU on the ED of signals transmitted in SISO systems based on OFDM rate-adaptive, margin-adaptive, and combined rate- and margin-adaptive transmission approaches. The obtained results indicated that the sensing performance was significantly impaired by NU. To reach a better level of detection performance, in [48] the analysis of the ED of the signals transmitted in the SISO–OFDM systems using DT adaptations was extended. The results indicated that SS implemented as ED with DT adaptations can improve the detection performance of the OFDM signals impacted by NU. In [49], we propose an algorithm for simulating the ED process based on SLC in MIMO-OFDM systems. According to the developed algorithm, analyses of impact of the false alarm on detection probability for different operating parameters in the MIMO-OFDM communication systems have been performed.

The previous research showed that the implementation of MIMO transmissions has an impact on the ED process [9,27,36,37,38,39,40,41,42,43]. Although a number of issues has been considered in related works, a comprehensive analysis dedicated to the ED performance based on SLC technique in MIMO-OFDM systems is still missing. In this work, mathematical expressions defining the relationship between detection probability, false alarm probability, SNR, and the number of samples used for SLC-based ED in MIMO-OFDM systems were developed for the first time. Based on the developed mathematical expressions, the performance analysis of the ED method using SLC for the realization of SS in MIMO-OFDM-based systems is further presented. This analysis tackles the impact of different parameters (SNR, Tx-Rx branch combinations, modulation techniques, transmit power, and false alarm probability) on the ED efficiency of the SLC method in MIMO-OFDM systems. The presented analysis of the simulation results, therefore, provides fundamental insights into the performance boundaries of ED based on the SLC technique in MIMO-OFDM systems.

## 3. System Model and Energy Detection Principles

### 3.1. System Model

**Figure 2:**
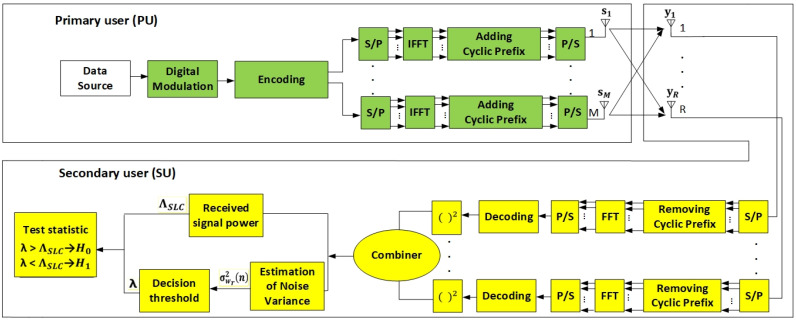
Block diagram of ED process based on SLC in a MIMO-OFDM system.

Figure 2 illustrates the analyzed MIMO-OFDM system composed of one PU and SU. After digital modulation and encoding, the signal is transmitted over each PU branch by means of OFDM, which includes serial to parallel (S/P) transform, Inverse Fast Fourier Transform (IFFT), cyclic prefix assignment, and P/S transform. On the SU side, the signal is received at each Rx antenna branch and OFDM demodulation is performed through the processes inverse to those of OFDM modulation. After this analog-to-digital conversion of the received signal at each Rx branch, the energy of the received signal at each branch is calculated and used to perform the SS based on ED.

In general, the PU has the priority when using a licensed spectrum. The SU as a cognitive user has the opportunity to use the licensed spectrum when it is not used by the PU. Hence, the SU must perform fast, accurate, and robust sensing of the spectrum state and obtain cognition in terms of the occupied or idle spectrum state. Depending on the spectrum state, which can be either unused or occupied by the PU signal, the signal detection of the SU can be modeled as a binary hypothesis testing problem. The hypotheses are marked as *H*_0_ and *H*_1_ to denote the PU signal absence or presence, respectively.

For simplicity, a system with one PU having *m* (*m* = 1,…, *M*) Tx branches (antennas) and one SU equipped with *r* (*r* = 1, …, *R*) Rx branches (antennas) was used in the analysis (Figure 2). It was further assumed that the PU transmits according to the main principles of the MIMO systems. This means that the data signals are transmitted over different Tx branches (antennas) at the same symbol rate using an equal carrier frequency. In the case of the PU transmission, the considered system is based on OFDM transmission (Figure 2). $P_{m}\;$ is assumed to be the Tx power allocated through the *m-th* antenna element, and the total instantaneous PU Tx signal power transmitted over the *M* Tx branches can be expressed as $\;P=\sum_{m=1}^{M}P_{m}.\;$ It is further assumed that the signal transmitted over the *m-th* Tx antenna denoted as $s_{m}$ is a complex signal ($s_{m}$ *= s_m,r_ + js_m,i_*).

All signals transmitted by the PU from the *M* Tx branches can be expressed as $s=\sum_{m=1}^{M}s_{m}$ (Figure 2). If the received signal at the *r-th* Rx branch (antenna) of SU is sampled with *n* samples (*n* = 1, …, *N*), then the received signal at each of the *R* Rx antennas of SU can be expressed as

(1) $$y_{r}\left(n\right)=\{\begin{array}{c} \;\;\;\;\;\;\;\;\;\;\;\;\;\;\;\;w_{r}\left(n\right)\;\;\;\\ h_{r\;}\left(n\right)\;s_{r}\left(n\right)+w_{r}\left(n\right)\;\; \end{array}$$

The $h_{r\;}\left(n\right)\;$ is a complex vector of size ${\mathbb{C}}^{1XM}$ representing the channel gain between the *M* Tx branches (antennas) and *r-th* Rx branch (antenna). The $s_{r}\left(n\right)$ is a complex vector with size ${\mathbb{C}}^{MX1}$ of the Tx signal, whose reception is performed during the *n-th* sample. The $w_{r}\left(n\right)$ is the complex noise sample at the *r-th* Rx branch (antenna). It is assumed to be a Gaussian random variable with zero mean and variance $\sigma_{w}^{2}$, which is characterized by a circular symmetric distribution $\;N\;\left(0,\;2\sigma_{w_{r}}^{2}\left(n\right)\right)$.

The signal $y_{r}$*(n*) detected at the *r-th* Rx branch of SU can be modeled as an unknown deterministic signal since it is assumed to be Gaussian with variance $2\sigma_{w_{r}}^{2}\left(n\right)$ and mean 𝔼 [$y_{r}$*(n)*] = 𝔼 [$h_{r\;}\left(n\right)\;s_{r}\left(n\right)+w_{r}\left(n\right)$]. Therefore, the expression of SNR at the *r-th* antenna of the SU can be given as

(2) $$SNR_{r}\left(n\right)=\gamma_{r}\left(n\right)=\frac{{\left|h_{r}\left(n\right)\right|}^{2}\;\frac{1}{N}\;\sum_{n=1}^{N}{\left|\;s_{r}\left(n\right)\right|}^{2}}{2\sigma_{w_{r}}^{2}\left(n\right)}$$

The SNR associated with the all *M* Rx antenna branches at the moment of the *n-th* sample is expressed as $\gamma_{SLC}=\sum_{r=1}^{R}\gamma_{r}\left(n\right)$, and the average SNR detected at the location of the SU for all *M* Rx antenna branches will be $\bar{\gamma_{SLC}}=\frac{1}{R}\sum_{r=1}^{R}\gamma_{r}\left(n\right)$=$\frac{1}{R}\gamma_{SLC}$.

If the received signal at all *R* of Rx branches (antennas) of SU is sampled with *n* samples (*n* = 1, …, *N*), then the received signal of the SU can be expressed as

(3) $$Y\;\left(n\right)=\{\begin{array}{r} \overset{R}{\underset{r=1}{\sum }}w_{r}\left(n\right):\;\;H_{0}\;\\ \overset{R}{\underset{r=1}{\sum }}h_{r\;}\left(n\right)\;s_{r}\left(n\right)+\overset{R}{\underset{r=1}{\sum }}w_{r}\left(n\right):\;\;H_{1} \end{array}$$

According to relation (1), *H*_1_ represents the presence of the PU signal and *H*_0_ denotes the null hypothesis, which indicates that there is no PU signal. In Table 1, all parameters used in the analysis with their corresponding descriptions are presented.

### 3.2. Energy Detection

For the purpose of the estimation of the ED performance, SLC as one of the prominent SL diversity methods was taken into consideration. The SLC is a non-coherent SS approach that exploits the diversity gain without the need for any channel state information. The digital implementation of energy detectors based on SLC in SISO and SIMO systems is able to obtain test statistics for energy detectors after applying filtering, sampling, squaring, and the integration of the received signal. The outputs of the integrator in SLC-based energy detection are known as the test (or decision) statistics. However, in MISO and MIMO systems, a device performing energy detection based on SLC must perform the squaring and integration operations for each diversity branch (Figure 2).

Following a square-law operation at each Rx branch, the SLC device combines the signals received at each Rx branch. The energy detector based on SLC finally receives the sum of the *R* test statistics (Figure 2), which can be expressed as follows.

(4) $$\Lambda_{SLC}=\overset{R}{\underset{r=1}{\sum }}\Lambda_{r}=\overset{R}{\underset{r=1}{\sum }}\overset{N}{\underset{n=1}{\sum }}{\left|y_{r}\left(n\right)\right|}^{2}\;$$

where $\Lambda_{r}$ represents the test statistics of the *r-th* Rx branch of the SU device.

It was shown in [32,41] that $\Lambda_{r}$ has a demanding distribution complexity. It involves non-central, chi-square distribution, which can be represented as a sum of the 2*N* squares of the independent and non-identically distributed (i.n.i.d.) Gaussian random variables with a non-zero mean. However, it is possible to reduce the distribution complexity through approximations by exploiting the central limit theorem (CLT) [32]. According to CLT, the sum of *N* independent and identically distributed (i.i.d) random variables with a finite variance and mean reaches a normal distribution when there is a sufficiently large N. Therefore, the approximation of the test statistic distribution $\Lambda_{SLC}$ (given in Equation (4)) can be performed using a normal distribution for an appropriately large number of samples *N* in order to be [32,41].

(5) $$\Lambda_{SLC}\sim N\left(\begin{array}{c} \overset{R}{\underset{r=1}{\sum }}\overset{N}{\underset{n=1}{\sum }}E\left[{\left|y_{r}\left(n\right)\right|}^{2}\right],\;\;\;\\ \overset{R}{\underset{r=1}{\sum }}\overset{N}{\underset{n=1}{\sum }}Var\left[{\left|y_{r}\left(n\right)\right|}^{2}\;\right]\; \end{array}\right)\;$$

where Var [ · ] and E [ · ] represent the variance and expectation operations, respectively. The variance and mean of the test statistics presented in Equation (5) under hypotheses $H_{0}$ and $H_{1}$ can be given as follows:

(6) $$\overset{R}{\underset{r=1}{\sum }}\overset{N}{\underset{n=1}{\sum }}Var\left[{\left|y_{r}\left(n\right)\right|}^{2}\right]=\{\begin{array}{r} \overset{R}{\underset{r=1}{\sum }}\overset{N}{\underset{n=1}{\sum }}[{\left(2\sigma_{w_{r}}^{2}\left(n\right)\right)}^{2}]:\;H_{0}\\ \overset{R}{\underset{r=1}{\sum }}\overset{N}{\underset{n=1}{\sum }}\left[4\sigma_{w_{r}}^{2}\left(n\right)\left(\sigma_{w_{r}}^{2}\left(n\right)+{\left|\;h_{r}\left(n\right)\right|}^{2}\;{\left|\;s_{r}\left(n\right)\right|}^{2}\right)\right]:\;H_{1} \end{array}$$

(7) $$\overset{R}{\underset{r=1}{\sum }}\overset{N}{\underset{n=1}{\sum }}E\left[{\left|y_{r}\left(n\right)\right|}^{2}\right]=\{\begin{array}{r} \overset{R}{\underset{r=1}{\sum }}\overset{N}{\underset{n=1}{\sum }}[2\sigma_{w_{r}}^{2}\left(n\right)]:\;H_{0}\\ \overset{R}{\underset{r=1}{\sum }}\overset{N}{\underset{n=1}{\sum }}\left[2\sigma_{w_{r}}^{2}\left(n\right)+{\left|\;h_{r}\left(n\right)\right|}^{2}\;{\left|\;s_{r}\left(n\right)\right|}^{2}\right]:\;H_{1} \end{array}$$

Assuming the constant channel gain $h_{r}\left(n\right)$ and nose variance $2\sigma_{w_{r}}^{2}\left(n\right)$ of the signal received at each of *R* of Rx antennas within each spectrum-sensing period *n*, the channel gain and noise variance can be expressed as:

(8) $$h_{r}\left(n\right)=h\;,\;\;\;\;\forall r=1,\;\ldots ,\;R;\;\forall n=1,\;\ldots ,\;N$$

(9) $$2\sigma_{w_{r}}^{2}\left(n\right)=2\sigma_{w}^{2}\;,\;\;\;\;\;\;\forall r=1,\;\ldots ,\;R;\;\forall n=1,\;\ldots ,\;N$$

Therefore, the SNR at *r-th* Rx branch (antenna) can be defined from relation (4) as:

(10) $$\gamma_{r}\left(n\right)=\gamma_{r}=\frac{{\left|\;h\right|}^{2}\frac{1}{N}\;\sum_{n=1}^{N}{\left|\;s_{r}\left(n\right)\right|}^{2}}{2\sigma_{w}^{2}}\;\;$$

In the case of a satisfactory number of samples, the variance of signal can be expressed as sample variance:

(11) $$2\sigma_{s_{r}}^{2}\left(n\right)\approx \frac{1}{N}\;\overset{N}{\underset{n=1}{\sum }}{\left|\;s_{r}\left(n\right)\right|}^{2}-{\left|\frac{1}{N}\overset{N}{\underset{n=1}{\sum }}s_{r}\left(n\right)\right|}^{2}$$

When the sample mean reaches zero $(\frac{1}{N}\;\sum_{n=1}^{N}s_{r}\left(n\right)\to 0$), the sample variance can be expressed as $2\sigma_{s_{r}}^{2}\left(n\right)=\frac{1}{N}\sum_{n=1}^{N}{\left|\;s_{r}\left(n\right)\right|}^{2}$.

Since ED as an SS method is characterized with no deterministic knowledge about the transmitted signal besides the average received power at the location of the SU, it can be assumed that the total instantaneous Tx power of the PU corresponds to the variance of all signals received at *R* of the Rx antennas such that $P=\sum_{r=1}^{R}{\left|\;h\right|}^{2}2\sigma_{s_{r}}^{2}\left(n\right)$. The relationship between the average SNR at the location of the SU and the average Tx power of the PU can be then approximated as $\bar{\gamma_{SLC}}\approx \frac{P}{R2\sigma_{w}^{2}}$.

Taking into account these assumptions, the distribution of the received signal test statistics $\Lambda_{SLC}$ can be expressed as follows.

(12) $$\Lambda_{SLC}\sim \{\begin{array}{r} N\;\left(RN\left(2\sigma_{w}^{2}\right),\;RN{\left(2\sigma_{w}^{2}\right)}^{2}\right):\;\;H_{0}\;\\ N\;\left(N\left(2\sigma_{w}^{2}\right)\left(R+\gamma_{SLC}\right),\;N{\left(2\sigma_{w}^{2}\right)}^{2}\left(R+2\gamma_{SLC}\right)\right):\;\;H_{1} \end{array}$$

By selecting each variance and mean presented in Equation (12), an approximated detection and false-alarm probability for SS based on ED using the SLC in MIMO OFDM systems can be derived.

### 3.3. Detection and False Alarm Probabilities

The probability of detection and false alarm are two important performance measures of any SS method including ED. The probability of false alarm ($P_{fa}$) is defined as the probability of detecting a PU signal at the location of the SU when the PU signal is not actually present. It is verified through the fulfillment of hypothesis *H*_0_ ($P_{f}[\mathrm{Pr}(\Lambda_{SLC}>\lambda )\;\cdot H_{1}]),\;$ where λ represents the detection threshold. For ED using the SLC approach in MIMO systems. It can be expressed as

(13) $$P_{f}[\mathrm{Pr}(\Lambda_{SLC}>\lambda )\;\cdot H_{0}]\approx Q\left(\frac{\lambda -RN\left(2\sigma_{w}^{2}\right)}{\sqrt{RN}\;\left(2\sigma_{w}^{2}\right)}\right)\;$$

In relation (13), Q is the Gaussian-Q function ($Q\left(x\right)=\frac{1}{\sqrt{2\pi }}\int_{x}^{\infty }e^{-\frac{u^{2}}{2}}$). According to Equation (13), an increase in false alarm probability reduces the spectrum usage by the SU and negatively impacts SS performance.

Detection probability ($P_{d}$) is the probability of detecting the PU signal at the location of the SU when it is actually present. It is verified through the fulfillment of hypothesis $H_{1}$ ($\left[\mathrm{Pr}(\Lambda_{SLC}<\lambda )\;\cdot H_{1}\right]$). For ED using the SLC approach in MIMO systems, detection probability can be expressed as

(14) $$\begin{array}{c} P_{d}[\mathrm{Pr}(\Lambda_{SLC}<\lambda )\;\cdot H_{1}]\approx Q\left(\frac{\lambda -N\left(2\sigma_{w}^{2}\right)\left(R+\gamma_{SLC}\;\right)}{\sqrt{N\left(R+2\gamma_{SLC}\right)}\;\left(2\sigma_{w}^{2}\right)}\right)\;\\ \approx Q\left(\frac{\lambda -RN\left(2\sigma_{w}^{2}\right)\left(1+\bar{\gamma_{SLC}}\;\right)}{\sqrt{RN\left(1+2\bar{\gamma_{SLC}}\right)}\;\left(2\sigma_{w}^{2}\right)}\right)\approx Q\left(\frac{\lambda -RN\left(2\sigma_{w}^{2}\right)\left(1+\frac{P}{2R\sigma_{w}^{2}}\;\right)}{\sqrt{RN\left(1+\frac{P}{{R\sigma }_{w}^{2}}\right)}\;\left(2\sigma_{w}^{2}\right)}\right) \end{array}$$

According to Equation (14), a higher detection probability increases the spectrum usage and positively impacts the SS performance of the SU. The approximations presented in relations (13) and (14) have been utilized to investigate the impact of SNR, a total number of samples (*N*), and the PU Tx power *P* of the detection probability. By combining relations (13) and (14), the relationship between the false alarm probability and detection probability can be expressed as

(15) $$P_{d}=Q\left(\frac{Q^{-1}\left(P_{f}\right)-\sqrt{\frac{N}{R}}\gamma_{SLC}}{\sqrt{\left(1+2\frac{\gamma_{SLC}}{R}\;\right)}\;}\right)=Q\left(\frac{Q^{-1}\left(P_{f}\right)-\sqrt{RN}\;\bar{\gamma_{SLC}}}{\sqrt{\left(1+2\;\bar{\gamma_{SLC}}\right)}\;}\right)=\;Q\left(\frac{Q^{-1}\left(P_{f}\right)-\frac{\sqrt{N}P}{2\sqrt{R}\sigma_{w}^{2}}}{\sqrt{\left(1+\frac{P}{R\sigma_{w}^{2}}\right)}\;}\right)\;$$

Based on relations (13) and (14), it can be noticed that the detection and false alarm probability depends on the level of the defined detection threshold. Additionally, the number of Rx branches (antennas), the SNR at the location of the SU, the overall number of samples during signal detection, and the variance of the noise all have influence on the SLC ED performance in MIMO systems. Additionally, from relation (15) it can be seen that the PU overall Tx power *P* has an influence on the probability of detection of the PU signal at the location of the SU.

### 3.4. Detection Threshold

As presented in relations (13) and (14), for the practical implementation of ED based on SLC, defining the operating detection threshold λ is required to obtain a decision regarding the absence or presence of a PU signal. Finding solutions for the optimal selection of a detection threshold is one of the main research interests in the field of SS. Different approaches to detection threshold selection have been proposed. They include the dynamic adaptation of the DT according to the instantaneous variations of the level of noise variations, through to setting the fixed threshold based on predefined parameters such as the constant false alarm probability. For example, the IEEE 802.22 systems specify targeted false alarm probability in order to be $P_{fa}<0.1$ [32]. Based on the given false alarm probability, the number of Rx branches and the noise variance, the expression-defining detection threshold in SLC ED systems is given in (13):

(16) $$\lambda_{f}=\left(Q^{-1}\left(P_{f}\right)+\sqrt{RN}\right)\sqrt{RN}2\sigma_{w}^{2}\;$$

Nevertheless, such a defined threshold cannot ensure that the energy detector based on SLC will obtain the minimal detection probability (which, in example of the IEEE 802.22 systems, is $P_{d}>0.9$ [32]). Hence, the selection of the detection threshold should maximize the detection probability and minimize the false alarm probability. It can be viewed as an optimization problem that must ensure a balance between the two conflicting objectives. For this reason, different approaches related to the improvement of detection performance are based on DT adaptation. The adaptation is performed according to the dynamic selection of the detection threshold, which can be in the range $\left[\frac{1}{\rho^{\prime }}\lambda ,\;\rho^{\prime }\lambda \right]$. Parameter $\rho^{\prime }$ represents the quantification parameter, which defines the range used for the dynamic selection of the threshold values.

### 3.5. Number of Samples

To achieve the requirements of the expected false alarm and detection probabilities, an important parameter in the SS process is the number of samples (*N*) used by the SLC energy detector during the detection of the PU signal. From relations (13) and (14), the minimum number of samples (*N*) can be found for the specified detection probability, the false alarm probability, the SNR, and the number of Rx branches (*R*). The minimum number of samples is not a function of the detection threshold and can be expressed as

(17) $$N=\frac{{\left[\sqrt{R}Q^{-1}\left(P_{f}\right)-\sqrt{\left(R+2\gamma_{SLC}\right)}Q^{-1}\left(P_{d}\right)\right]}^{2}}{\gamma_{SLC}{}^{2}}$$

$$=\frac{{\left[Q^{-1}\left(P_{f}\right)-\sqrt{\left(1+2\bar{\gamma_{SLC}}\right)}Q^{-1}\left(P_{d}\right)\right]}^{2}}{R{\bar{\gamma_{SLC}}}^{2}}$$

From relation (17), it can be seen that $O(1/{\bar{\gamma_{SLC}}}^{2})$ is the order of the approximate number of samples N needed to obtain the predefined detection and false alarm probabilities.

Additionally, the $Q^{-1}$(.) function has a monotonical decreasing behavior. This ensures that an increase in the number of samples during SS can guarantee the detection of signals with very low SNRs in the case where there is perfect knowledge of the noise power. However, if the number of samples increases, the sensing duration also increases. This is the main drawback of the ED method based on SLC, since, at low SNRs, a large number of samples is needed for accurate detection. Increasing the sensing duration can be problematic in terms of its practical implementation because some systems have a specified maximal sensing duration (for example, for IEEE 802.22 systems, maximal sensing duration is 2 s [32]). An increased sensing time has a negative impact in terms of the battery depletion of power-constrained devices such as sensors and other devices working in the IoT environment. The selection of the number of samples used for ED is also an optimization challenge.

### 3.6. Noise Variance

According to relations (13) and (14), the noise variance ($\sigma_{w}^{2})$ has a strong influence on the selection of the detection threshold and, consequently, on the detection and false alarm probability. According to relation (16), finding an appropriate detection threshold can be done only when the noise variance (power) $\sigma_{w}^{2}$ is perfectly known at the SU.

Due to impacts such as temperature variations, interference, and filtering effects, perfect knowledge of the noise variance in practice is not always possible. As a consequence, the information about the properties of the AWGN can be limited and this contributes to the presence of errors in the noise power estimation. This is known as NU and this phenomenon can significantly impair the performance of ED based on the SLC. When NU exists, the interval $\left[\frac{1}{\rho }\sigma_{w}^{2},\;\;\rho \sigma_{w}^{2}\right]$ can be assumed to be an interval that quantifies the range of NU variations, where ρ (ρ>1) represents the quantification parameter. In this paper, the analysis was performed while considering the impact of NU on ED performance.

To illustrate the impact of low SNR on the selection of the number of samples *N* that can ensure ED, in (17) a low SNR can be approximated as $1+\bar{\gamma_{SLC}}\approx 1$. To achieve the specific false alarm and detection probabilities, the needed number of samples for the SLC-based energy detector can be expressed as

(18) $$N=\frac{{\left[\sqrt{R}Q^{-1}\left(P_{f}\right)-\sqrt{R}Q^{-1}\left(P_{d}\right)\right]}^{2}}{{\left(\bar{\gamma_{SLC}}-\left(\rho -\frac{1}{\rho }\right)\right)}^{2}}$$

According to relation (18), achieving the target detection and false alarm probability can be accomplished only if an infinitely large number of samples ($\bar{\gamma_{SLC}}$→$\left(\rho -\frac{1}{\rho }\right)$) is used for the ED. Since ED based on SLC cannot work at such a level, this drawback is defined as the *SNR* wall phenomenon. The SNR wall defines the lowest SNR value for which ED can be performed using a specific number of samples (*N*), while considering the detection and false alarm probabilities.

## 4. Algorithm for Simulating Energy Detection

The algorithms developed for simulating the ED process in MIMO-OFDM CRNs are presented in this section. The simulation of ED performance is performed in two phases. In the first phase, the generated *MxR* MIMO-OFDM signal transmitted by the PU with the implementation of the MIMO-OFDM signal reception is presented with Algorithm 1. Additionally, in the second phase, the simulation of the SLC ED process impacted by NU fluctuations and performed by exploiting the DT adaptation is modeled using the pseudocode of Algorithm 2.
**Algorithm 1.** Generation of *m×r* MIMO OFDM signals.*1: **Input 1:** Number of transmit antennas (m=M), number of Rx antennas (r=R), modulation order K (QPSK, 16 QAM, 64 QAM), number of samples (N), frame size (framelen), length of cyclic prefix (cp_len), range of SNR simulated values (SNR_loop), number of transmitted packets in each simulation run (packets number), the overall number of channels (L)**,** reference constellation (refconst), normalization type (type), and Tx power (power).* *2:**Output:** Received MIMO OFDM signal (mimo_ofdm_received_signal_M×r)* *3: **Initialize**: **Input1*** *4:                         **FOR** i = 1: SNR_loop;* *5:                         SNR = SNR_loop (i);* *6:                         NPW = 10^(-SNR/10);* *7:                         **FOR** i = 1: packets number;* ***Step 1:****Generate vector of random data points for K-PSK or K-QAM modulation* *8:*                             *x = randint*
*(N, framelen, K);*

*9:                             Scale=modnorm (refconst, ‘type’, power);*
10:                            S_Usr1=Scale*psk(qam)mod(x, K);

***Step 2:***
*Perform transmission with STBCs*
*11:                      *
*X=*
*S_Usr1*
*[:, framelen];*

***Step 3:***
*Perform IFFT*

*12:                       S_t_m= ifft(****X****);*

***Step 4:***
*Compute Cyclic Prefix;*

*13:                       S_t_cp_m= [ S_t_m (end-cp_len+1: end,:); S_t_m ];*

***Step 5:*** *Parallel to serial transformation*

14:                     s_tx_m= reshape(S_t_cp_ m, 1, framelen*(N + cp_len));

***Step 6:***
*Set*
*channel transmission coefficients with fading*

*15:                      *
*h_mr =*
1/sqrt(2*M*(L+1))*randn(1,L+1);
***Step 7:*** *Generation of transmitted signal in multipath channel*
*16:                       s_rx_r = 0;*

*17: **FOR**                    l = 1:L+1*
*18:                       s_rx_r = s_rx_r +*
h_mr*s_tx_m;

*19: **END***
***Step 8:*** *Impact fo noise on transmitted signal*
20:                      n_r = (NPW/2)*randn(1, length(s_rx_r));
*21:                      s_rx_r_n = s_rx_r + n_r;*
***Step 9****: Reception of signal at r-th branch of SU*

*22:            **FOR** r= 1:R*
*23:           **FOR** k = 1:framelen*
24:           S_M×r = [s_rx_r_n ((N + cp_len)*(k-1)+1:(N + cp_len)*k) ];
*25:           S_M×r_cp_r = S_M×r (cp_len + 1:end,:);*

*26:           S_M×r_f_r = fft(S_M×r_cp_r);*

*27:           **END***
*28:           **END***
***Step 10****: FFT estimation of chanel matrix coeffcients*

*29:                       h_f_ M×r= fft([h_mr zeros(1,N-(L+1))].’);*
***Step 11:***
*Reception of signal at r-th branch after OFDM demodulation*
*30:                       FOR p = 1:N*

*31:                      H = [h_f_ M×r (p)];*

*32:                      r_p = [S_ M×r _f_r (p,:)];*
33:                      mimo_ofdm_received_signal_M×r= r_p*H
*34:                          **END***

*35:                          **END***

*36:                          **END***


### 4.1. Algorithm for Simulating MIMO-OFDM Signal Generation and Reception

Algorithm 1 shows the details of the pseudocode dedicated to the generation of the MIMO-OFDM signal used for the assessment of ED performance. Algorithm 1 enables the generation of different MIMO-OFDM-modulated signals (64 QAM, 16 QAM, and QPSK) for the purpose of the simulations.

The first line of Algorithm 1 shows the setup of the input parameters, based on which the generation of the MIMO-OFDM signals will be performed. The values including the overall number of PU Tx antennas (*M*), the overall number of SU Rx antennas (*R*), the modulation order *K* (64 QAM, 16 QAM, and QPSK), the number of samples (*N*), the frame size (*framelen*), the length of OFDM cyclic prefix (*cp_len*), the range of analyzed SNR values (*SNR_loop*), the number of transmitted packets (*packets number*), the total number of channels used for transmission (*L*), the reference constellation (*refconst*), the normalization types (*type*), and the Tx power (*power*) are set.
**Algorithm 2.** ED process based on SLC for *M*×*R* MIMO-OFDM system.*1: **INPUT:** mimo_ofdm_received_signal_M×r,number of samples (N), SNR_loop, DT factor (ρ′), NU factor (ρ), noise variance (*$\sigma_{n_{i}}^{2})$*, range of*$P_{fa_{i}}$*and number of Monte Carlo simulations (kk)* *2: **OUTPUT:** Probability of detection (*$P_{d_{i}}^{NUDT})$ *3: **ON INITIALIZED** Received MIMO-OFDM signal (mimo_ofdm_received_signal_M×r) do:* ***Step 1****: Simulation of detection probability (*$P_{d}$*)* vs. *SNR based on (14), (15)*

*4:               set kk = number of Monte Carlo simulations*

*5:               set SNR_loop = signal to noise ratio [−25, 10]*
*6:   **FOR**         p = 1:length (SNR_loop)*
*7:*                   *i1= 0;*

*8:   **FOR***
*i = 1:10, 000;*
***Step 2:***
*Modeling the impact of NU on the received signal*
*9:             Noise uncertiaity (*ρ
*>1.00) = sqrt(*$\sigma_{w_{r}}^{2}\left(n\right)>1.00$).*randn (1, framelen);
*10:           received_signal_M×r*
*= mimo_ofdm_received_signal_M×r*
*+ Noise uncertainty;*
***Step 3:***
*Received signal energy calculation based on SLC*
*11:             **REPEATE FOR r= 1:R***
*12:       energy_calc_r*
*= abs(received_signal_M×r).^2;*

*13: **END***
***Step 4:***
*Test statistic calculation based on combining energies of R signals (based on (4))*

*14:                        **FOR r= 1:R***
*15:       test_stat = sum(energy_calc_r);*

*16: **END***
***Step 5:***
*Threshold evaluation (based on (12))*
*17:           thresh (p) = ((qfuncinv(*$P_{fa}$
(p)).* ρ./sqrt(N))+ ρ)./ $\rho^{\prime }$*;*
***Step 6:***   *Decision making process*

*18:                   **IF** (test_stat >= thresh (p));*

*19:                     i1 = i1 + 1;*
*20:                   **END***

*21:       **END***

***Step 7:***
*Monte Carlo simulation-determining*
$P_{d}$
*(based on (1))*
*22:      *
$P_{d_{i}}$
*(p) = i1/kk;*

*23:         **END***

*24:         **UNTIL***
$P_{d_{i}}$*= [0, 1]*

In Algorithm 1, lines 3–7, the simulated SNR range (lines 4–5), the SNR normalization-to-linear scale (line 6), and the number of packets used in the simulation (line 7) are initialized. In lines 8–10, a random data points’ vector consisting of *K-PSK-* or *K-QAM-*modulated signals is generated, and defining the scaling factor for the Tx power output normalization is committed. In line 11, the process of generating an encoded signal is performed. The encoding process is performed for the *M* OFDM transmit branches (Figure 2). Line 12 presents the application of an inverse fast Fourier transform (*ifft*) to each block of OFDM signal for the *m = M* transmit branches (antennas). The CP computation and appending of CP to every OFDM block on each Tx antenna is performed in line 13. A parallel to the serial transformation of the OFDM signal for transmission over each PU antenna is performed in line 14.

Modeling the wireless channel impacted with fading is presented in line 15 of Algorithm 1. Lines 16–19 present the generated MIMO-OFDM signals transmitted using the encoded signal (*s_rx_r)* in the multipath channel. Pseudocode lines 20–21 of Algorithm 1 present the modeling of the impact of AWGN (*n_r*) on the transmitted signals (*s_rx_r_n).*

The reception of the MIMO-OFDM signal at the location of the SU having *r = R* Rx branches is modeled in lines 22–28 (Figure 2). The signal reception is modeled in line 22 for each Rx antenna and for each ODDM symbol in line 23. Signal reception includes the serial-to-parallel conversion (modeled in line 24), removing the CP (modeled in line 25) and performing the fast Fourier transform (*fft*) of the received signal (modeled in line 26).

In line 29, the calculation of the different transmission coefficients *h_f_* *M×r* of the channel matrix *H* is performed.

Depending on the total number of samples (*p = 1:N*), in line 30, the reception of the signal for every *N* samples is executed. In line 31, the calculation of the channel matrix *H* is based on transmission coefficients *h_f_* *M×r*, and this is performed for each sample *N.* Additionally, for each sample *N*, the signal at each Rx antenna (*S_M×r_f_r*) is modeled in line 32 (Figure 2). Finally, pseudocode line 33 shows the calculation of the final OFDM *Mxr* signal received at each of the *R* SU antennas (*mimo_ofdm_received_signal_* *M×r*). This signal is used as the input signal for Algorithm 2.

### 4.2. Algorithm for Simulating Energy Detection in MIMO-OFDM System Based on SLC

The first line of Algorithm 2 indicates the setup of the input parameters used for simulating the ED process. The parameters, including the received MIMO-OFDM signal (*mimo_ofdm_received_signal_M×r*), the number of samples (*N*), the SNR simulation range(*SNR_loop)*, the NU factor (ρ), the DT factor ($\rho^{\prime }$), the noise variance ($\sigma_{n_{i}}^{2}$), the range of false alarm probabilities ($P_{fa}$), and the overall size of Monte Carlo simulations (*kk*), are set.

In lines 4–8 of Algorithm 2, the total number of Monte Carlo simulations for a specific SNR range are defined and executed. In line 9, the level of NU is defined in the form of the NU factor (ρ > 1.00), and in line 10, the impact of the defined NU level on the received MIMO signal is modeled for each Rx branch.

Lines 11–16 model the ED process based on the SLC of the received MIMO signal. The energy of the received signal at each individual Rx branch (antenna) is calculated in pseudocode lines 11–12 (Figure 2). The operation of combining energies of the received signals detected at each of the *R* Rx antennas is performed in lines 14–15. The result of this process represents the MIMO-OFDM signal test statistics (*test_stat)* received at the location of the SU (Figure 2).

Line 17 presents the estimation of the received signal threshold (*thresh(p)*) using the process of DT adaptation based on the defined DT factor $\rho^{\prime }$. The decision-making process in terms of the PU signal energy presence or absence is presented in lines 18–21 of Algorithm 2 (Figure 2). If the received signal energy is larger than or the same as the threshold, then the PU is present and *H*_1_ hypothesis is validated. If the received signal energy is lower than the threshold, then the PU is absent and hypothesis *H*_0_ is validated. In lines 22–24, the large number of Monte Carlo iterations are executed in order to obtain an appropriate simulation accuracy. For each SNR value, the detection probability of the PU signal is calculated in order to be expressed in the range of 0–1 (Table 2).

## 5. Simulation Results

In this section, the parameters used in simulations and analyses of simulation results are presented. Spectrum sensing based on the ED technique in MIMO-OFDM CRNs was simulated for the SISO and symmetric and asymmetric MIMO transmissions. The signal transmission was impaired by NU variations, and signal detection was performed based on the DT adaptations. The differences between the received PU signals in terms of the Tx power, the number of samples, the different modulation types, and the target false alarm probabilities were simulated for both the SISO and versatile MIMO transmission concepts.

### 5.1. Simulation Software and Parameters

The modeling of the SS based on the SLC ED method in MIMO-OFDM CRNs and generating the MIMO-OFDM signal according to Algorithm 1 was performed using Matlab software (version R2016a). Developed Matlab code was executed according to the pseudocode of Algorithm 1 directly from the Matlab editor. Additionally, to simulate the ED process exploiting the SLC technique, the same principles based on execution of developed Matlab code defined with pseudocode of Algorithm 2 were performed. Table 2 lists all the parameters used in the simulations.

As shown in Table 2, a different number of PU Tx and SU Rx branches were used in the simulations. Additionally, 64 QAM, 16 QAM, and QPSK types of OFDM modulations, which are frequently used in the real implementations of OFDM-based systems, were used in the simulations. Additionally, Table 2 indicates that, in the analysis, a versatile number of samples (1024, 512, 256, and 128) for the detection of OFDM signals were used. The SNR range of the received signals selected for analysis was between −25 dB and 25 dB (Table 2). This SNR range corresponds to the operating environments of a large number of communication technologies that use OFDM transmissions.

The validation of the simulation results was performed for 10,000 Monte Carlo simulations (Table 2) per each simulating MIMO-OFDM configuration. This number of Monte Carlo simulations was selected for ensuring the trade-off between the simulation duration and simulation accuracy. In total, 48 different MIMO-OFDM configurations (operation scenarios) were analysed (Table 2). The considered configurations represented typical operational environments that characterise practical implementations of the MIMO-OFDM system. Such a number of MIMO-OFDM configurations and Monte Carlo simulations per each configuration ensured that the performed simulation analyses were realistic.

To simulate realistic PU signal detection, the NU factor was set to 1.02 and the DT factor was set to a value of 1.01 (Table 2). The selection of such NU and DT factors reflects a simulation with a moderate impact of NU and an appropriate level of DT adoption.

Table 3 presents the execution time of Algorithm 2 for each of the 48 simulated configurations characterized with corresponding configuration parameters. Additionally, for all simulated configurations, Table 3 contains information about maximal, minimal, mean, and median values of the execution time of Algorithm 2. According to the data presented in Table 3, the execution time of Algorithm 2 ranged from 13 µs to 61 µs, with an average and median execution time for all 48 simulation configurations equal to 24.27 µs and 22 µs, respectively. Based on the obtained results for execution time, which was calculated using time measuring functions in Matlab software, the execution time of Algorithm 2 was fast and was applied in practical implementations.

The computational time of Algorithm 2, which is presented in Table 3, was low, due to the low computational complexity of the energy detection based on the ED with SLC method. In comparison with other prominent local spectrum-sensing methods such as Waveform Based, Cyclostationary Feature Detection, and Matched Filter, the ED method has lower computational complexity [48]. This is a consequence of a higher amount of prior information about PU, which, in the case of these methods, needed to be delivered to the PU for performing successful spectrum sensing. This consequence, with fundamental principles of the SLC ED process, which are based on combining energies of signals received at each Rx branch of SU, is the main reason for the fast execution of Algorithm 2.

### 5.2. Impact of SISO and MIMO Transmission on the ED Performance

**Figure 3:**
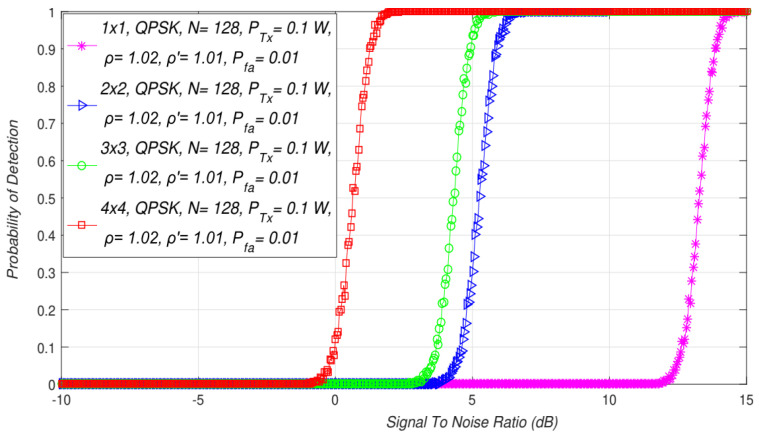
Interdependence between the probability of detection and SNR for SISO and different symmetric MIMO Tx-Rx branch combinations.

The results of the first simulation study presented the impact of SISO and the symmetric MIMO on the ED performance (Figure 3). The analysis was performed for a constant PU Tx power (0.1 W). The presented results were obtained for SISO (1 × 1) and different symmetric MIMO combinations of the Tx and Rx branches (2 × 2, 3 × 3, 4 × 4), a constant quantity of OFDM samples (128), and the target probability of a false alarm ($P_{fa})$ equal to 0.01.

The results presented in Figure 3 indicate that for the larger levels of SNR, the detection probability was enhanced for any MIMO Tx-Rx branch combination. The detection probability decreases with the degradation of SNR. However, to obtain a better detection probability of the PU signal, the results presented in Figure 3 indicate that involving a transmission with multiple Tx and Rx branches at the PU and SU, respectively, can improve the detection probability.

According to the presented simulation results in Figure 3, the PU signal detection probability will be improved for higher SNRs and a higher number of transmitting/receiving branches at both the PU and SU sides. A higher number of Tx branches on the PU and Rx branches at the SU enables more accurate signal detection due to the diversity effect on the signal transmission. This improves the ED process and contributes to the increase in the PU detection probability for systems with a higher number of Tx and Rx branches at both the PU and SU sides, respectively.

**Figure 4:**
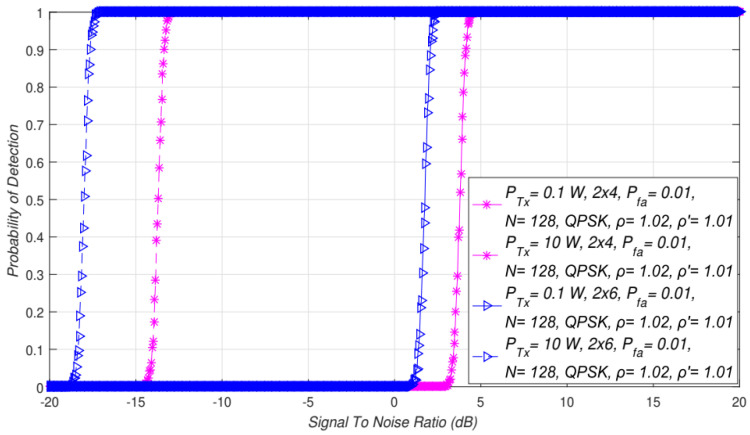
Interdependence between the probability of detection and SNR for different asymmetric MIMO Tx-Rx combinations and PU Tx powers.

In Figure 4, interdependence between the probability of detection and SNR for different asymmetric MIMO Tx-Rx combinations and PU Tx powers is presented. The results presented in Figure 4 indicate that for the same Tx power and SNRs, the detection probability was higher for the systems with more antennas at the receiver side (2 × 6 MIMO in comparison with 2 × 4 MIMO). This is a consequence of the larger diversity at the Rx side (SU), where the larger number of Rx branches (antennas) can process the larger number of PU signal copies and their corresponding energies. This led to the conclusion that a higher Tx power and a larger number of antennas at the SU side (Figure 4) gives significant contribution to the enhancement of the detection probability in the case when NU factor, DT factor, the number of samples *N*, and the target false alarm probability $P_{fa}$ are predefined.

### 5.3. Impact of the Number of Samples on the ED Performance in MIMO-OFDM Systems

**Figure 5:**
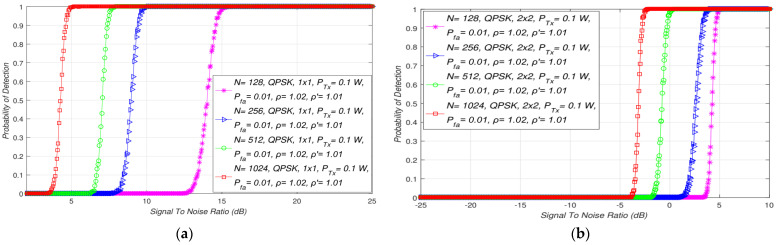
Influence of the number of samples on the detection probability for: (**a**) SISO and (**b**) symmetric MIMO transmission systems.

The second test performed was dedicated to the analyses of the influence of the number of samples on the SLC ED performance in SISO and MIMO-OFDM CRNs. In Figure 5a,b, the interdependence between detection probability ($P_{d}$) and SNR for different numbers of samples (*N*) in SISO and symmetric MIMO-OFDM systems is presented. The simulation results were obtained for the SISO and 2 × 2 MIMO-OFDM systems and for the predefined false alarm probability equivalent to $P_{fa}$ = 0.1, constant Tx power (100 mW), fixed NU and DT factors (Table 2), and modulation constellation (QPSK).

According to the results presented in Figure 5, a high influence on the ED performance in the MIMO-OFDM systems had the number of samples used during the ED. The obtained results presented in Figure 5 showed that for any number of Tx-Rx branch combinations, the detection probability enlarged when a larger number of samples during the ED process was used. This is a consequence of a higher number of samples used for ED, which results in a higher number of signal detection attempts during a specific sensing period in which the signal of the PU is trying to be detected.

Additionally, the results obtained indicated that a worse detection probability is acquired for a lesser SNR and a lower number of samples in the case of any Tx-Rx branch configurations (Figure 5). Since a lesser SNR is a direct consequence of the influence of the stronger nose impacting the PU signal transmission, the detection probability will decline as the value of SNR decreases. Furthermore, Figure 5 shows the existence of the SNR threshold, and for SNR values lower than this threshold, the detection probability cannot be guaranteed ($P_{d}$ < 100%). The level of the SNR threshold will be higher for signals detected with a higher number of samples or with the MIMO-OFDM configurations with a larger number of Tx-Rx branches (Figure 5b).

Therefore, increasing the detection probability is related to the trade-off between the number of samples used during the ED process and the number of Tx and Rx branches (antennas) of both the PU and SU, respectively. According to this trade-off, Figure 5 shows that, for a higher number of Tx-Rx branches and a lower number of samples, it is possible to obtain similar detection probabilities as those obtained in the systems with a lower number of Tx-Rx branches (antennas) and a larger sample quantity.

This confirms that the detection probability enlarges when a higher number of Tx-Rx branches (antennas) and number of samples are used during the ED (Figure 5). Hence, MIMO systems outperform SISO systems in terms of ED performance based on the SLC in the case when the analysis was performed for the same number of samples, Tx powers, and channel characteristics. This is due to the transmission diversity characteristic for the MIMO transmissions, which contribute to the improvement of the ED performance in the case of a lower number of samples.

### 5.4. Impact of the Different Modulations on the ED Process in MIMO-OFDM Systems

**Figure 6:**
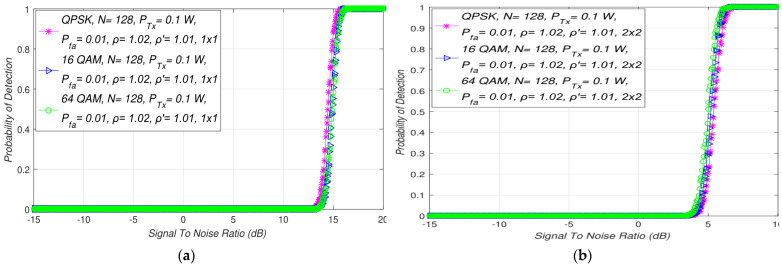
Interdependence between detection probability and SNR for ED with different modulation techniques in: (**a**) SISO and (**b**) symmetric MIMO transmission systems.

The presented simulation results in this section are directed to studying the influence of the OFDM modulation type and the modulation order (constellation) on the capability of PU detection during the ED process. The results presented in Figure 6 showed that for the predefined levels of PU Tx power (0.1 W), the number of samples (*N*), the values of false alarm probabilities $\left(P_{fa}\right),$ and the NU and DT factors, the detection probability will remain unchanged for any modulation. This confirms that there is no direct impact on detection probability at any SNR level of any OFDM modulation and corresponding constellation. If an equal Tx power is used for transmission of the differently modulated OFDM signals in the same channel impacted by the same NU detected using the same number of samples with an equal DT factor, the ED performance based on the SLC will remain unchanged for every OFDM signal (Figure 6).

Although the modulation order does not have any effect on the PU signal detection, from Figure 6b it can be seen that MIMO-OFDM systems can enhance the ED process. More specifically, the obtained results showed that, for a higher number of Tx-Rx branches (Figure 6b), the probability of detection will increase in comparison to that of the SISO systems(Figure 6a). According to the presented results in Figure 6, the MIMO *2 × 2* system had an SNR wall for values of SNRs that were significantly better (3 dB) than those of the SISO system (13 dB). This means that the MIMO transmission resulted in a faster PU ED at the location of the SU. As a consequence, this improved the ED process.

### 5.5. Impact of Primary User Transmission Power on ED Performance

**Figure 7:**
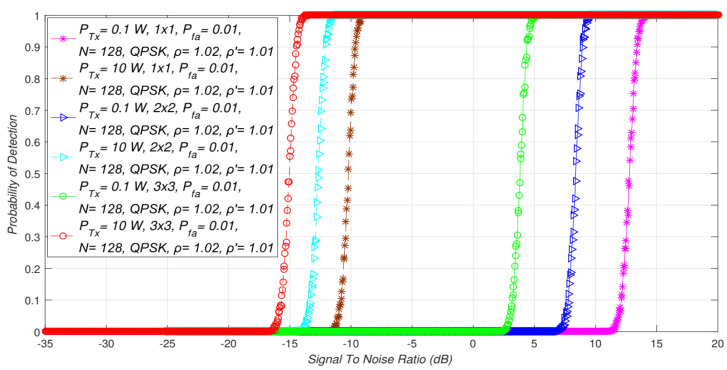
Interdependence between detection probability and SNR for the ED of the signals transmitted with different PU Tx powers in symmetric MIMO transmission systems.

In Figure 4, simulation results were obtained for two PU Tx powers (0.1 W and 10 W). The obtained results indicated the influence of asymmetric MIMO-OFDM transmissions (*2 × 4* and *2 × 6*) on ED performance. Additionally, in Figure 7, the impact of different PU Tx powers (0.1 W and 10 W) on ED performance in symmetric MIMO-OFDM systems (*1 × 1*, *2 × 2*, *3 × 3*) is presented. The results were obtained for the fixed NU factor, DT factor, the probability of a false alarm (Table 2), and the modulation order (QPSK).

The results presented in Figure 4 and Figure 7 showed that for the same SNR values, a higher detection probability will be present when it comes to sensing the MIMO-OFDM signals transmitted at higher Tx powers. This was confirmed for the ED of the signals transmitted in symmetric (Figure 7) and asymmetric (Figure 4) MIMO configurations. This result was expected since the higher PU Tx power induces a larger amount of the PU signal received at the location of the SU. This larger energy will be received for any combination of Tx and Rx branches involved in the signal transmission and detection.

Additionally, the results presented in Figure 4 and Figure 7 showed that transmission with a higher Tx power and a larger number of Tx-Rx branches had a positive influence on the level of SNR walls. Therefore, a combination of the PU Tx power level, the number of Tx-Rx branches, and the SNR at the location of the SU has a dominant impact on the ED performance in terms of detection probability. For the larger SNRs, the higher Tx power levels, and the MIMO systems having more Tx-Rx branches, the detection probability will be larger and vice versa. For environments with low SNRs, the detection probability at the location of the SU can be increased by combining the transmission at a higher Tx power with the enlargement of the Tx-Rx branches.

### 5.6. Impact of False Alarm Probabilities on the ED Performance in MIMO-OFDM Systems

**Figure 8:**
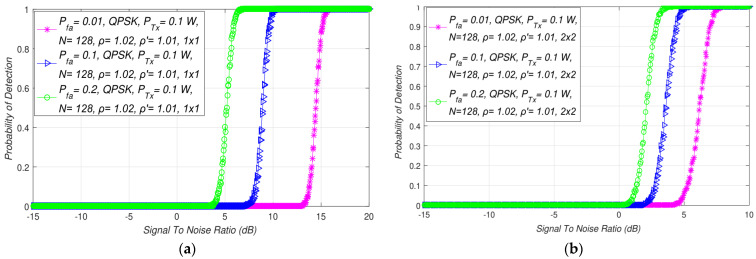
Interdependence between detection probability and SNR for different values of false alarm probability in: (**a**) SISO and (**b**) a symmetric MIMO transmission system.

The simulation results presented in this section are focused on the overview of the influence of different false alarm probabilities on the detection probability in MIMO-OFDM CRNs. The interdependence among detection probability and SNRs for distinct false alarm probabilities $(P_{fa}=0.01,\;0.1,\;0.2$) and specified fixed Tx power (*P =* 0.1 W), the number of samples (*N* = 128), QPSK modulation, the NU (ρ=1.02), and the DT factors ($\rho^{\prime }=1.01$) in SISO and symmetric MIMO (2 × 2) systems are presented in Figure 8a,b, respectively. Since the false alarm probability should be as low as possible, up to 20% of a false alarm probability can be accepted in real implementation. The analysis performed for different false alarm probabilities can simulate the small ($P_{fa}=1\%$), moderate $\left(P_{fa}=10\%\;\right),$ and large $(P_{fa}=20\%$) influence of NU on the ED performance.

The results presented in Figure 8 pointed out that the detection probability will be larger if the false alarm probability is larger and vice versa. The reduction in the false alarm probability was followed by a reduction in the detection probability for the SISO (Figure 8a) and symmetric MIMO (Figure 8b) systems. This is a consequence of the fact that when a real PU exists and the SU correctly estimates this existence, the possibility that the SU incorrectly deduces that a PU is present starts to rise. This causes a decline in the false alarm probability accompanied by a decrease in the detection probability, and vice versa.

However, by comparing the results presented in Figure 8a,b, it can be noticed that for the same false alarm probability, the SNR, PU Tx power, and the NU detected using the equal DT adaptation factor, the detection probability will be higher for MIMO transmissions. An SLC-based ED process aimed at ensuring a constant false alarm rate (CFAR) can, therefore, benefit from MIMO transmission since CFAR can be obtained by increasing the number of Tx-Rx branches in the environments characterised with large SNRs.

It can be further seen in Figure 8 that the MIMO transmission and higher false alarm probability increased the detection probability of the PU signals in OFDM-based systems. Nevertheless, it should be considered that the enlargement in the probability of false alarms enlarged the possibility of incorrect decisions made by the SU during the ED. For that reason, an adequate trade-off among the PU Tx power, the number of Tx-Rx MIMO branches, and the minimal false alarm probability must take place. This further proves the importance of MIMO transmission. In this transmission, the number of Tx-Rx branches represents the additional variables, which must be included into the decision-making process dedicated to the improvement of the ED performance for any value of demanded CFAR.

### 5.7. Impact of the Number of Transmitting and Receiving MIMO Branches on ED Performance

**Figure 9:**
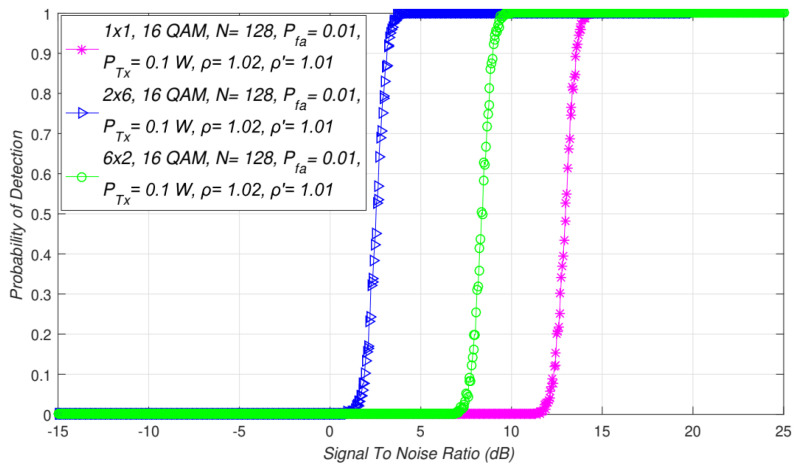
Interdependence between detection probability and SNR for SISO and 2 × 6 and 6 × 2 asymmetric MIMO transmission system.

Further simulation results that were analysed presented the influence of SISO (1 × 1) and asymmetric MIMO- OFDM (*MxR, M* ≠ *R*) transmission on the performance of ED based on SLC. In Figure 9, the simulation results for the ED performance of SISO and 6 × 2 and 2 × 6 asymmetric MIMO-OFDM systems are presented. The results were obtained for the specific PU Tx power (0.1 W), the modulation type (16 QAM), the number of samples(*N =* 128), the false alarm probability (0.01), and the specified NU (1.02) and DT (1.01) factors.

In comparison with the SISO systems, Figure 3, Figure 7 and Figure 8 show that symmetric MIMO-OFDM transmission systems for any combination of Tx-Rx branches will yield better ED performance. The results presented in Figure 9 additionally confirm this conclusion for ED performed with any combination of Tx-Rx branches in asymmetric MIMO transmission systems. More specifically, any combination of an unequal number of Tx-Rx branches in the MIMO-OFDM system contributes to the improvement of ED performance based on the SLC. The results presented in Figure 9 show that, in comparison with SISO systems, asymmetric MIMO systems obtain the same probability of detection for lower SNR values.

Compared to SISO systems, the SNR walls (in dB) are, therefore, significantly lower for ED in systems with an unequal (asymmetric) number of Tx-Rx MIMO branches (Figure 8). This is a consequence of the positive impact that diversity transmission brings into the ED process. Even for an unequal number of Tx-Rx branches with at least one side of the communication link having a number of branches larger than one, asymmetric MIMO systems will have a better ED performance than SISO systems.

**Figure 10:**
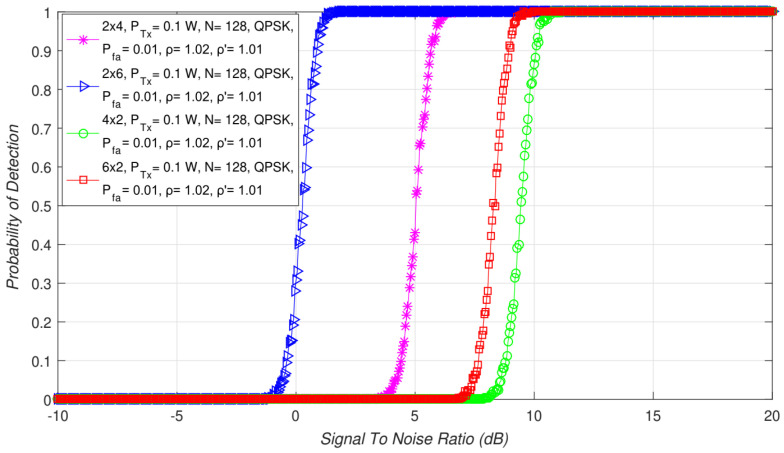
Interdependence between detection probability and SNR for different combinations of asymmetric Tx-Rx branches of MIMO transmission systems.

Additionally, a comparison of the ED performance between different asymmetric MIMO-OFDM systems with distinct combinations of Tx-Rx branches (6 × 2, 2 × 6, 4 × 2, 2 × 4) is presented in Figure 10. According to the results presented in Figure 10, the number of Tx and Rx branches used for transmission and ED in asymmetric MIMO-OFDM systems has a non-negligible impact on the ED performance. The obtained results showed that higher detection probabilities will be accomplished if the SS is performed with a higher number of Tx and Rx branches (better ED performance for 6 × 2 in comparison with *4 × 2* MIMO systems). This was also confirmed for ED in the symmetric MIMO-OFDM systems, as presented in Figure 2 (better ED performance for 4 × 4 in comparison with 2 × 2 MIMO systems).

However, the results presented in Figure 10 showed that a more positive impact on ED performance will be if the number of Rx branches is larger than the number of Tx branches (better ED performance for 2 × 6 in comparison with 6 × 2 MIMO systems). To further emphasize the importance of the number of Rx branches in the ED process, the results presented in Figure 10 showed that, even for 2 × 4 systems, the ED performance will be better than it is for 6 × 2 MIMO-OFDM systems. These results are the consequence of the stronger contribution that the larger number of Rx branches brings to the ED process.

Since the SLC method is based on combining the energy of the signals received at each Rx branch, a larger number of Rx branches positively impacts ED performance.

### 5.8. Impact of the False Alarm on Detection Probability for Different SNRs

**Figure 11:**
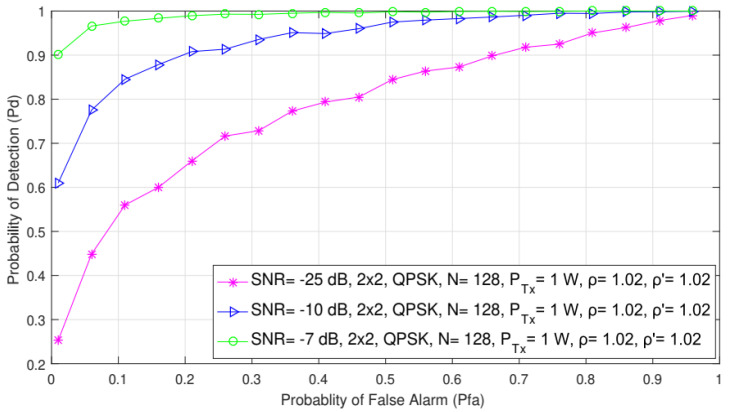
Interdependence between detection and false alarm probabilities for different SNR values.

In Figure 11, the impact of different probabilities of the false alarm on detection probability is presented for three SNR levels (−25, −10, and −5 dB). The results were obtained for the fixed PU Tx power (1 W), the constant number of samples (N = 128), the QPSK modulation, the symmetric 2 × 2 MIMO-OFDM systems, and the NU and DT factors, $\rho^{\prime }=1.01$ and ρ=1.02, respectively.

According to the results shown in Figure 11, the probability of detection is reduced for lower levels of SNR (−25 dB) at the location of SU. Hence, obtained results again confirmed that the value of SNR at the location of SU had a direct impact on detection probability. As the SNR values increase (−10, −5 dB), the detection probability will also be improved. This improvement of PU signal detection is a direct result of a higher SNR level, which results in higher energy of the signal for the same PU Tx power at the location of SU.

**Figure 12:**
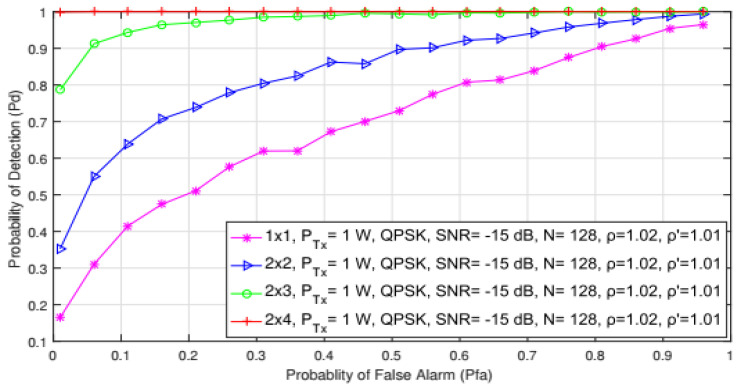
Interdependence between detection and false alarm probabilities for the SISO and symmetric and asymmetric MIMO-OFDM systems.

In Figure 12, the impact of different probabilities of the false alarm on detection probability for the SISO and the symmetric (2 × 2) and asymmetric MIMO (2 × 3 and 2 × 4) systems are shown. Analyses were performed for transmission with the QPSK modulation, the number of samples (N = 128), the constant values of SNR (−15 dB), and PU Tx power of 1 W.

The results presented in Figure 12 showed that for the same values of false alarm probability ($P_{fa}$), detection probability increased with a higher number of Tx-Rx branches on the PU and SU sides (results for 2 × 2 MIMO were significantly better in comparison with those obtained for SISO). Additionally, detection probability increased as the number of Rx antennas increased in asymmetric MIMO systems (results for 2 × 4 were significantly better in comparison with the 2 × 3 system). This is a consequence of the higher number of Rx branches, which enable the processing of a higher number of PU signal copies. Consequently, this improved the detection probability of PU signals. These results were fully in line with those presented in Figure 10, where a higher number of Rx antennas also had a positive impact on ED performance. More Rx branches mean more energy at the location of SU and better PU signal detection.

Therefore, the impact that the increased number of Tx branches had on the ED performance was lower than the impact that the increased number of Rx branches had on the overall ED process. Therefore, the ED in the MIMO-OFDM system will benefit from the future generation of SU devices whose practical implementation will generally involve a larger number of antennas and corresponding communication branches.

## 6. Conclusions

In this paper, the performance analysis of ED as a low-complexity SS method based on square-low combining is presented. Tests were performed for CRNs containing one PU and SU that employed MIMO-OFDM transmissions. To perform the analysis, a mathematical formulation indicating the dependence of the PU signal detection probability on a set of parameters including the false alarm probability, the number of samples used for ED, the number of PU Tx and SU Rx branches, the SNRs at the location of the SU and PU Tx powers was developed. The expressions were derived for MIMO-OFDM systems exploiting the diversity of the transmissions over a different number of PU Tx and SU Rx branches. The simulation study was performed based on the algorithms developed for simulating the process of transmitting signals in the MIMO-OFDM systems and the SS of said signals using the SLC ED method. To conduct a realistic performance analysis, the algorithm proposed for simulating the ED process incorporated the impact of the adaptations of DT and NU variations during the ED process.

A study of the results obtained through extensive simulations presented how the SISO and MIMO transmissions, the number of samples used for SS, the different modulations, the PU Tx powers, the false alarm probabilities, and the number of PU Tx and SU Rx MIMO branches impacted ED performance in MIMO-OFDM systems. Besides the different modulation types, it was shown that the other analyzed parameters had a non-negligible impact on the overall ED performance. It was shown that significant improvements in ED performance can be achieved through the appropriate selection of analyzed parameters. They can include some or all of the activities related to the increase in PU Tx power, the number of PU Tx and especially SU Rx branches, the number of samples used for ED and probabilities of false alarms. The presented results and analysis can, therefore, serve as the basis for the development of new or improved SS approaches using the SLC ED method in MIMO-OFDM systems.

Future research activities will be focused on studying the influence of the different levels of NU variations and the DT adaptations of the ED process based on the SLC in MIMO-OFDM systems.

## Figures and Tables

**Table 1:** Parameters used in the analyses.

Index	Description
$H_{1}$	The hypothesis that determines the presence of the PU signal
$H_{0}$	The hypothesis that determines the absence of the PU signal
*m*	Number of PU Tx branches (antennas)
*r*	Number of SU Rx branches (antennas)
*M*	Total number of transmit antennas at the PU
*R*	Total number of receiving antennas at the SU
*N*	Total number of samples used in the detection process
$P_{m}$	Transmit (Tx) power allocated through the *m-th* antenna element of the PU
*P*	Total instantaneous Tx power of the PU transmitted over the *M* Tx branches
$s_{m}$	The complex signal transmitted over the *m-th* Tx antenna of the PU
s	The overall complex signal transmitted by the PU from the *M* Tx branches
$y_{r}\left(n\right)$	The received signal at the *r-th* Rx branch (antenna) of the SU during the *n-th* spectrum-sensing period
Y(n)	The overall signal received at the *R* Rx branches (antennas) of the SU during the *n-th* spectrum-sensing period
$h_{r\;}\left(n\right)$	$\mathrm{Channel}\;\mathrm{gain}\;\mathrm{between}\;\mathrm{the}\;M\mathrm{Tx}\;\mathrm{antennas}\;\mathrm{and}\;\mathrm{the}\;r\mathrm{th}\;\mathrm{Rx}\;\mathrm{branch}\;(\mathrm{complex}\;\mathrm{vector}\;\mathrm{of}\;\mathrm{size}\;{\mathbb{C}}^{1XM}$) during the *n-th* spectrum-sensing period
$s_{r}\left(n\right)$	$\mathrm{Signal}\;\mathrm{vector}\;{\mathbb{C}}^{MX1}$ received during the *n-th* sample at the *r-th* Tx branch (antenna)
$w_{r}\left(n\right)$	Complex noise vector at the *r-th* Rx branch (antenna) of the SU in the *n-th* spectrum-sensing period
$\sigma_{w_{r}}^{2}\left(n\right)$	Nose variance of the signal detected at the *r-th* Rx antenna of the SU in the *n-th* spectrum-sensing period
$\sigma_{s_{r}}^{2}\left(n\right)$	Received signal variance at the *r-th* Rx branch (antenna) of the SU in the *n-th* spectrum-sensing period
$\gamma_{r}\left(n\right)$	SNR at the *r-th* antenna of the SU in the moment of the *n-th* spectrum-sensing period
$\gamma_{SLC}\left(n\right)$	Total SNR associated with the *M* Rx antenna branches in the moment of the *n-th* spectrum-sensing period
$\bar{\gamma_{SLC}}\left(n\right)$	Average SNR detected at the location of the SU device for all *R* Rx antenna branches in the *n-th* spectrum-sensing period
$\Lambda_{r}$	Test statistics of the signals received over the *r-th* Rx branch (antennas) of the SU device
$\Lambda_{SLC}$	Total test statistics of the signals received over the *R* Rx branches (antennas) of the SU device
Var [ · ]	Variance operation
E [ · ]	Expectation operation
$P_{f}$	False alarm Probability
$P_{d}$	Detection probability
Q(x)	Gaussian-Q function
λ	Detection threshold
$\lambda_{f}$	False alarm detection threshold in the SLC ED systems
*ρ*	NU factor
$\rho^{\prime }$	DT factor
*L*	Number of channels used for transmission

**Table 2:** Simulation parameters.

Parameters	Type/Quantity
Transmission type of PU signal	OFDM
Number of transmit antennas	1–4
Number of receive antennas	1–6
Type of OFDM (constellation)	QPSK, 16 QAM, 64 QAM
Channel noise type	AWGN
Quantity *N* of samples (FFT size)	128, 256, 512, 1024
The range of SNRs at location of SU (dB)	−25–25
The detection and false alarm probabilities’ range	0–1
No. of Monte Carlo iterations/simulation	10,000
NU factor *ρ*	1.02
DT factor *ρ*′	1.01
Target False alarm probability	0.01, 0.1, 0.2
Total number of analysed MIMO-OFDM Tx-Rx configurations	48

**Table 3:** Execution time of Algorithm 2 for different simulated MIMO-OFDM configurations.

Sim. Conf. No.	Simulation Configuration: No. of Tx/Rx Branches, Tx Power $\left(P_{Tx}\right)$, False Alarm Probability $\left(P_{fa}\right)$ Number of Samples (*N*), Modulation Type, NU/DT Coefficients $\left(\rho /\rho^{\prime }\right)$	Exec. Time (µs)	Sim. Conf. No.	Simulation Configuration: No. of Tx/Rx Branches, Tx Power $\;(P_{Tx})$, False Alarm Probability $\left(P_{fa}\right)$, Number of Samples (*N*), Modulation Type, NU/DT Coefficients $\rho /\rho^{\prime }$	Exec. Time (µs)
1.	*1 × 1*, 0.1 W, 0.01, 128, QPSK, 1.02/1.01	18	29.	*1 × 1*, 0.1 W, 0.01, 128, QPSK, 1.02/1.01	15
2.	*2 × 2*, 0.1 W, 0.01, 128, QPSK, 1.02/1.01	16	30.	*1 × 1*, 0.1 W, 0.1, 128, QPSK, 1.02/1.01	19
3.	*3 × 3*, 0.1 W, 0.01, 128, QPSK, 1.02/1.01	18	31.	*1 × 1*, 0.1 W, 0.2, 128, QPSK, 1.02/1.01	26
4.	*4 × 4*, 0.1 W, 0.01, 128, QPSK, 1.02/1.01	19	32.	*2 × 2*, 0.1 W, 0.01, 128, QPSK, 1.02/1.01	22
5.	*2 × 4*, 0.1 W, 0.01, 128, QPSK, 1.02/1.01	24	33.	*2 × 2*, 0.1 W, 0.1, 128, QPSK, 1.02/1.01	28
6.	*2 × 4*, 10 W, 0.01, 128, QPSK, 1.02/1.01	22	34.	*2 × 2*, 0.1 W, 0.2, 128, QPSK, 1.02/1.01	21
7.	*2 × 6*, 0.1 W, 0.01, 128, QPSK, 1.02/1.01	28	35.	*1 × 1*, 0.1 W, 0.01, 128, 16 QAM, 1.02/1.01	18
8.	*2 × 6*, 10 W, 0.01, 128, QPSK, 1.02/1.01	22	36.	*2 × 6*, 0.1 W, 0.01, 128, 16 QAM, 1.02/1.01	15
9.	*1 × 1*, 0.1 W, 0.01, 128, QPSK, 1.02/1.01	17	37.	*6 × 2*, 0.1 W, 0.01, 128,16 QAM, 1.02/1.01	19
10.	*1 × 1*, 0.1 W, 0.01, 256, QPSK, 1.02/1.01	33	38.	*2 × 4*, 0.1 W, 0.01, 128, QPSK, 1.02/1.01	21
11.	*1 × 1*, 0.1 W, 0.01, 512, QPSK, 1.02/1.01	41	39.	*2 × 6*, 0.1 W, 0.01, 128, QPSK, 1.02/1.01	22
12.	*1 × 1*, 0.1 W, 0.01, 1024, QPSK, 1.02/1.01	61	40.	*4 × 2*, 0.1 W, 0.01, 128, QPSK, 1.02/1.01	29
13.	*2 × 2*, 0.1 W, 0.01, 128, QPSK, 1.02/1.01	17	41.	*6 × 2*, 0.1 W, 0.01, 128, QPSK, 1.02/1.01	22
14.	*2 × 2*, 0.1 W, 0.01, 256, QPSK, 1.02/1.01	23	Sim. Conf. No.	Simulation Configuration: No. of Tx/Rx Branches, Tx Power $\left(P_{Tx}\right)$, SNR, Number of Samples (N), Modulation Type, NU/DT Coefficients $\rho /\rho^{\prime }$	Exec. time (µs)
15.	*2 × 2*, 0.1 W, 0.01, 512, QPSK, 1.02/1.01	34			
16	*2 × 2*, 0.1 W, 0.01, 1024, QPSK, 1.02/1.01	50			
17.	*1 × 1*, 0.1 W, 0.01, 128, QPSK, 1.02/1.01	28			
18.	*1 × 1*, 0.1 W, 0.01, 128, 16 QAM, 1.02/1.01	20	42.	*2 × 2*, 1W, −25 dB, 128, QPSK, 1.02/1.01	49
19.	*1 × 1*, 0.1 W, 0.01, 128, 64 QAM, 1.02/1.01	22	43.	*2 × 2*, 1 W, −10 dB, 128, QPSK, 1.02/1.01	18
20.	*2 × 2*, 0.1 W, 0.01, 128, QPSK, 1.02/1.01	21	44.	*2 × 2*, 1 W, −7 dB, 128, QPSK, 1.02/1.01	34
21.	*2 × 2*, 0.1 W, 0.01, 128, 16 QAM, 1.02/1.01	29	45.	*1 × 1*, 1 W, −15 dB, 128, QPSK, 1.02/1.01	45
22.	*2 × 2*, 0.1 W, 0.01, 128, 64 QAM, 1.02/1.01	22	46.	*2 × 2*, 1 W, −15 dB, 128, QPSK, 1.02/1.01	15
23.	*1 × 1*, 0.1 W, 0.01, 128, QPSK, 1.02/1.01	14	47.	*2 × 3*, 1 W, 15 dB, 128, QPSK, 1.02/1.01	28
24.	*1 × 1*, 10 W, 0.01, 128, QPSK, 1.02/1.01	16	48.	*2 × 4*, 1W, −15 dB, 128, QPSK, 1.02/1.01	14
25.	*2 × 2*, 0.1 W, 0.01, 128, QPSK, 1.02/1.01	18		Minimal duration of execution time	13
26.	*2 × 2*, 10 W, 0.01, 128, QPSK, 1.02/1.01	25		Maximal duration of execution time	61
27.	*3 × 3*, 0.1 W, 0.01, 128, QPSK, 1.02/1.01	14		Average duration of execution time	24.27
28.	*3 × 3*, 10 W, 0.01, 128, QPSK, 1.02/1.01	13		Median duration of execution time	22

## Abbreviations

The following abbreviations are used in this manuscript:

AWGN	Additive white Gaussian noise
BS	Base station
CFAR	Constant false alarm rate
CLT	Central limit theorem
CP	Cyclic prefix
CR	Cognitive radio
CRN	Cognitive radio networks
CSI	Channel state information
CSS	Cooperative spectrum sensing
DSA	Dynamic spectrum access
DT	Dynamic threshold
ED	Energy detection
EGC	Equal Gain Combining
IoT	Internet of Things
ISI	Inter-symbol interference
MIMO	Multiple-input multiple-output
MISO	Multiple input-single output
MRC	Maximal Ratio Combining
NU	Noise uncertainty
OFDM	Orthogonal frequency-division multiplexing
PU	Primary user
RF	Radio frequency
ROC	Receiver operating characteristic
SISO	Single-input single-output
SIMO	Single-input multiple-output
SL	Square-law
SLC	Square-law combining
SLS	Square-Law Selection
SNR	Signal-to-noise ratio
SS	Spectrum sensing
STBC	Space–time block codes
SU	Secondary users
